# Cross-cultural adaptation and validation of the Physical Therapy Outpatient Satisfaction Survey in an Italian musculoskeletal population

**DOI:** 10.1186/1471-2474-14-125

**Published:** 2013-04-05

**Authors:** Carla Vanti, Francesca Bonetti, Daniele Ceron, Raffaella Piccarreta, Francesco Saverio Violante, Andrew Guccione, Paolo Pillastrini

**Affiliations:** 1 Department of Biomedical and Neuromotor Sciences (DIBINEM), Alma Mater Studiorum, University of Bologna, Bologna 40138, Italy; 2 Associazione Italiana Riabilitazione Inserimento Invalidi, Rehabilitation Center, Via Novacella 40, Rome 00142, Italy; 3 Riabilita Physical Therapy, Masters in Manual Therapy and Musculoskeletal Rehabilitation, University of Padova, Padova 35121, Italy; 4 Department of Decision Sciences, L.Bocconi University, Milan 20100, Italy; 5 Department of Medical and Surgical Sciences (DIMEC), Alma Mater Studiorum, University of Bologna, Bologna 40138, Italy; 6 Department of Rehabilitation Science, College of Health and Human Services, George Mason University, Fairfax, VA 22030, USA; 7 Department of Biomedical and Neuromotor Sciences (DIBINEM) Alma Mater Studiorum, University of Bologna, Via Pelagio Palagi 9, Bologna 40138, Italy

**Keywords:** Health care administration, Measurement, Outcome measures

## Abstract

**Background:**

Although patient satisfaction is a relevant outcome measure for health care providers, few satisfaction questionnaires have been generally available to physical therapists or have been validated in an Italian population for use in the outpatient setting. The aim of this study was to translate, culturally adapt, and validate the Italian version of the Physical Therapy Outpatient Satisfaction Survey (PTOPS).

**Methods:**

The Italian version of the PTOPS (PTOPS-I) was developed through forward-backward translation, review, and field-testing a pre-final version. The reliability of the final questionnaire was measured by internal consistency and test-retest stability at 7 days. Factor analysis was also used to explore construct validity. Concurrent validity was measured by comparing PTOPS-I with a 5-point Likert-type scale measure assessing the Global Perceived Effect (GPE) of the treatment and with a Visual Analogue Scale (VAS).

**Results:**

354 outpatients completed the PTOPS-I, and 56 took the re-test. The internal consistency (Cronbach’s alpha) of the original domains (*Enhancers*, *Detractors*, *Location*, and *Cost*) was 0.758 for *Enhancers*, 0.847 for *Detractors*, *0.885* for *Location*, and 0.706 for *Cost*. The test-retest stability (Intra-class Correlation Coefficients) was 0.769 for *Enhancers*, 0.893 for *Detractors*, 0.862 for *Location*, and 0.862 for *Cost*. The factor analysis of the Italian version revealed a structure into four domains, named *Depersonalization, Inaccessibility*, A*mbience,* and *Cost*. Concurrent validity with GPE was significantly demonstrated for all domains except *Inaccessibility.* Irrelevant or non-significant correlations were observed with VAS.

**Conclusion:**

The PTOPS-I showed good psychometric properties. Its use can be suggested for Italian-speaking outpatients who receive physical therapy.

## Background

The quality of physical therapy treatment is measured on several factors, including patient satisfaction [1,2]. Satisfaction ratings may reflect a range of factors such as personal preferences of the patient, patient expectations, and the nature of the care received and services provided [3]. Responses to satisfaction surveys are difficult to interpret because they usually refer to a complex function of expectations that may vary greatly among patients despite comparable care [4]. However the use of feedback provided by patient satisfaction surveys helps to incorporate patient views into clinical practice and may lead to improved outcomes following treatment [5,6]. Over the years, several questionnaires have been developed to evaluate concepts such as patient satisfaction or experience [7-10]. Each of these instruments captures various aspects of the construct of ‘patient satisfaction’. Although there is no universal gold standard for measuring patient satisfaction [11], one of the most studied instruments on satisfaction with physical therapy is the MedRisk Instrument for Measuring Patient Satisfaction With Physical Therapy Care (MRPS) [11]. MRPS has a two-factor structure relating to external factors such as admissions and clinical environment and to an internal factor relating to the patient-therapist interaction. Both factors and all items within these two factors demonstrated high significant correlation with global measures of satisfaction [12].

The Physical Therapy Satisfaction Questionnaire (PTPSQ), developed by Goldstein, Elliott, and Guccione consists of 26 items, of which 20 items explore the interaction with the physical therapist and the staff and some environmental factors such as location, parking, cost [13]. The psychometric properties of the original version of the PTPSQ were tested on 289 subjects and indicated a one-dimension structure, dominated by satisfaction with the physical therapist interaction. Notably, opinions on the cost of the treatments appeared to be less related to the overall satisfaction.

Monnin and Perneger developed a 14-item instrument of patient satisfaction with physical therapy, applicable to both inpatient and outpatient settings [14]. This questionnaire measures satisfaction in 3 dimensions: treatment, admission, and logistics. It also contains a global assessment subscale. After administration to 528 Swiss patients, the validity analysis confirmed grouping the items into the 3 dimensions. Satisfaction scores appeared related to the patient’s intention to recommend the facility and to the number of positive and negative comments to open-ended questions.

The Physical Therapy Outpatient Satisfaction Survey (PTOPS) was developed in 1999 by Roush and Sonstroem [15] and was designed to address the multiple domains of patient satisfaction most often mentioned in the literature. The psychometric properties of the final version with 34 Likert-scale items were established on 173 patients across 4 domains, which the authors named *Enhancers*, *Detractors*, *Location*, and *Cost*. Specifically, the *Enhancers* domain concerned satisfaction with the physical environment and relationships inherent in a clinical situation and covers elements that enhance a positive patient experience above a minimally acceptable or basic level. In contrast, the items in the *Detractors* domain generate disappointment, but do not necessarily generate satisfaction when they are met. In this domain, perceptions regarding professional behaviors are particularly relevant. Items in the *Location* domain refer to the location of the facility, travel time, and easiness of reaching it. Finally, the *Cost* domain consists of items related to the balance between the perceived value of treatment and its actual cost.

A European-English version of the PTOPS, validated in Ireland, was published in 2007 [16]. Significant correlations were observed between the *Enhancers*, *Detractor*, and *Cost* domains and the responses to four global satisfaction questions, but no significant correlation was found with the *Location* domain.

To the authors’ knowledge, the PTOPS has never been validated in other languages than U.S. English and European English. As a consequence, we did not know if its psychometric characteristics would be confirmed in an Italian population. Several scales for pain, disability, function, and general health are widely employed to measure the outcome of the outpatient physical therapy in Italy, but no translated form of a ‘specialty-specific’ satisfaction questionnaire has been validated to date.

The aims of this study were to translate, culturally adapt, and investigate the psychometric properties (acceptability, reliability, and validity) of the PTOPS in an Italian population. We also examined relationships between the indicators of satisfaction and some characteristics of the physical therapists and of the patients.

## Methods

### Subjects

This study involved a Hospital-based outpatient clinic and two private outpatient physical therapy facilities, located in three different geographic areas representing different social and cultural contexts. All centers involved were specialized in musculoskeletal disorders.

All patients who attended these facilities from April 2011 to September 2011 were asked to participate, without any restriction related to the type of disease/disorder. Adults (18 years or older) who were able to read and speak Italian were included in the sample; patients who received only one treatment or those with cognitive and psychiatric conditions were excluded.

All subjects gave their written consent. The Ethic Committee of the University Hospital S. Orsola-Malpighi of Bologna (Italy) approved the trial (code 32/2011/U/OssN).

### Examiners

Research assistants administered the questionnaires. Moreover, subjects were assured that neither their physical therapists nor other team members would have knowledge of their answers.

The questionnaires were completed at the time of treatment and in separate rooms to respect patient privacy. Levels of understanding and acceptability were recorded for each question, and the time needed for the answers was noted for each subject.

### Translation and cross-cultural adaptation of the PTOPS

We followed the Guidelines for the Process of Cross-Cultural Adaptation of Self-Report Measures [17].

Step 1. Forward Translation. PTOPS was forward translated from English into Italian so as to retain the meaning of the questions in the original questionnaire. The first translations were independently done by two different native-speaking Italian translators. One translator (the ‘naïve’ translator) was not familiar with the topic. The translators tried to keep the language compatible with a reading age level of 14 years and geared to a low-level of education. When a concept had no equivalent in the Italian culture or language, it was modified to suit the cultural context. The choices about the most complicated items were resolved in person by the two translators. For the correct interpretation of item 29 (‘Facility appreciates my business’) the first author of the PTOPS was consulted. None of the items was omitted.

Step 2. Backward Translation. Two bilingual native English-speaking translators backward translated the first version to verify that the Italian version adhered to the sense of the original version as much as possible. The two translators were neither informed of the concepts being explored nor aware of the original version, and they did not have any medical backgrounds.

Step 3. Expert Committee. Both the forward and the backward translations were submitted to a bilingual committee, including four experts in physical therapy, a linguist and the four translators. To identify difficulties and to correct mistakes, the committee discussed various options for items and responses, with respect to semantic, idiomatic, experiential, and conceptual equivalence based on the original version. None of the items was omitted. This step ended when a satisfactory formulation of the beta version was obtained.

Step 4. Test of the Beta Version. The beta version was administered to 50 outpatients to verify if all the items and responses were understood correctly. All the results obtained in this phase were re-evaluated by the expert committee and some minor adjustments related to vocabulary were made. None of the items was omitted. At the end of this stage, an Italian version, PTOPS-I, was set for psychometric testing (see Additional file 1: PTOPS-I).

Step 5. Psychometric scale properties and data analyses.

### Procedures of administration

All subjects provided some information about their socio-demographic characteristics and the nature of their physical therapy visit (how and why they were referred, if it was their first treatment, etc.), and completed the PTOPS-I questionnaire. A brief introductory paragraph explained that the results would be anonymous, would not affect their treatment in any way, and that the physical therapists would not know how their patients answered the questionnaires. Furthermore, to investigate the concurrent validity, a scale evaluating the Global Perceived Effect (GPE) of the physical therapy treatment compared to their initial condition [18] and a Visual Analogue Scale (VAS) for the current assessment of their pain were administered [19-21]. The GPE questionnaire is a 5-point Likert-type scale evaluating the patient’s perception about the effectiveness of the received treatment. Subjects answered the question ‘How do you rate the overall effectiveness of the treatment with respect to your needs?’ and selected one of the five possible answers, ranging from ‘it really helped’ (score 1) to ‘it made things worse’ (score 5). The VAS scale is based on a line 10 cm long, whose extremes correspond to ‘absence of pain’ (score 0) and ‘unbearable pain’ (score 100). The patient is asked to quantify his/her perceived pain by identifying the point indicating the pain felt at the time of administration.

### Statistical analysis

#### Acceptability

The time needed from each subject to complete the questionnaire was recorded. Also the examiners registered possible difficulties or mistakes in the questionnaire compilation, and the number of missing answers, wrong or multiple responses.

#### Definition of domains

Roush and Sonstroem [21] defined their domains (hereafter, R-S-domains) based on factor analysis, which yielded a 4-factors solution.

We performed two separate analyses: the first one according to the R-S-domains extracted from the U.S. data with our own data and the second one to explore the possibility of different domains, specific to the Italian population. One single factor was extracted for each domain.

#### Reliability

Internal consistency was measured using Cronbach’s (1951) alpha (α) on the whole set of PTOPS-I items (0.7 < α < 0.8 = acceptable, 0.8 < α < 0.9 = good, α > 0.9 = excellent). If deletion of an item from a domain causes a decrease of α, this indicates that the item is actually strongly connected to the others, so that its deletion makes the domain less internally consistent. On the other hand, if the deletion of one item from a domain causes a consistent increase of the domain’s α, this indicates that the deleted item is weakly related to the others, so that its deletion makes the domain more internally consistent.

To explore the test-retest reliability, a sample of randomly selected outpatients filled out the Italian version of PTOPS again one week later. The Intra-class Correlation Coefficients (ICC) together with their 95% confidence intervals (0.6 < ICC < 0.8 = good reliability, ICC > 0.8 = excellent reliability) were used to evaluate the agreement between the test and the retest *total* scores (one for each domain). The same criteria were used to assess the item-by item agreement.

Construct validity was examined *for each domain* using factor analysis. Thus, factor analysis was applied to each domain separately to evaluate whether the assumption of a unique factor satisfactorily summarizing the items in each domain was supported by the data.

#### Concurrent validity

The concurrent validity was tested by correlating the PTOPS-I domain totals with a measure of pain, the Visual Analogue Scale (VAS), and one measure of global satisfaction, the Global Perceived Effect (GPE) of the physical therapy treatment using Pearson’s *r* (*r* < 0.30 = low; 0.30 < *r* < 0.60 = moderate; *r* > 0.60 = good).

It was hypothesized *a priori* that the correlation between the PTOPS-I domains’ totals and the GPE would be moderate to low and the correlation with the VAS would be low.

#### Analysis of dependence

We employed Kruskal-Wallis and Wilcoxon tests to investigate if, and to what extent, the domain totals depended on the characteristics of the therapy (i.e. the attended facility, the facility’s advisor, whether or not it was the first time that the patient attended the facility or had an episode of care by a physical therapist, regular attendance at therapy, the therapist’s gender and the combination of the therapist-patient genders, and the method of payment) as well as patient characteristics (i.e. gender, age class, marital status, education, working status), to investigate whether it is possible to individuate groups of patients being particularly satisfied or dissatisfied about the therapy.

#### Statistical methods

All statistical analyses were performed using PASW Statistics, 18 (Release 18.0.3) and SAS (Release 9.2).

## Results

### Subjects

357 subjects were asked to participate to this study. Only 3 subjects refused; therefore, the trial was based upon a sample of 354 subjects, with mean age 49.96 years (SD = 13.48). Complete information was available for 345 subjects. Nonetheless, for some domains a higher number of cases is available (since some of the 354 subjects provided complete information for one domain but not for another).

Socio-demographic characteristics of the sample are described in Table 1.

**Table 1:** Characteristics of the sample

**Variable**	**Category**	**n**	**Percentage**
**Gender**	Female	237	66.95%
	Male	117	33.05%
**Age (classes)**	18-25	12	3.39%
	25-40	62	17.51%
	40-65	242	68.36%
	>65	38	10.73%
**Married**	Yes*	232	65.54%
	No**	121	34.18%
	No answer	1	0.28%
**Workers**	Yes	268	75.71%
	No	29	8.19%
	Retired	56	15.82%
	No answer	1	0.28%
**Smokers**	Yes	76	21.47%
	No	278	78.53%
**Education**	Elementary	15	4.24%
	Mid school	71	20.06%
	Upper school	177	50.00%
	University	91	25.71%
**Duration of the dysfunction**	<1 month	16	4.52%
	1 month ≤ 3 months	41	11.58%
	> 3 months	297	83.90%
**Disease/Dysfunction**	Spinal pain	235	66.38%
	Other	119	33.62%
**Facility recommended by**	Doctor	62	17.51%
	Friends	49	13.84%
	Other Patients	39	11.02%
	Other	1	0.003%
	No answer	203	57.35%
**First treatment in the facility**	Yes	221	62.43%
	No	133	37.57%
**First episode of physical therapy care**	Yes	126	35.59%
	No	228	64.41%
**Regularly attends scheduled sessions**	Yes	317	89.55%
	No	37	10.45%
**Therapist’s gender**	Female	141	39.83%
	Male	213	60.17%
**Payment**	Direct payment	126	35.59%
	Co-Payment	2	0.56%
	Fully covered (National Health System)	79	22.32%
	Insurance	147	41.53%

* = married, living together. ** = single, widowed, divorced.

56 subjects repeated the completion of the questionnaire one week after the first administration to allow the analysis of test-retest stability.

### Translation and cross-cultural adaptation

The forward backward translation process performed by 4 translators required 3 months to achieve a culturally adapted version. A further revision and adaptation after the beta version testing required 1 month.

### Psychometric characteristics

#### Acceptability

**Figure 1:**
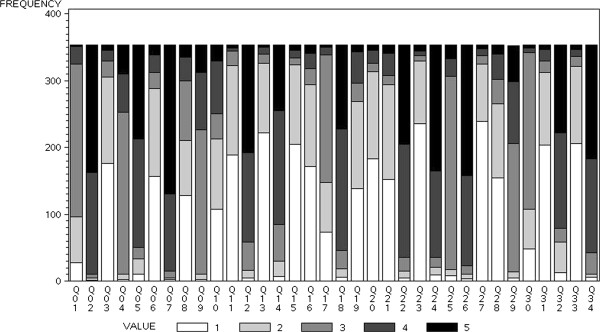
**Frequency distributions of the variables.** Each distribution is represented by stacked bars, each stack representing one of the possible values (1 = Strongly Disagree; 2 = Disagree; 3 = Uncertain; 4 = Agree; 5 = Strongly Agree) taken by the variable. The height of a stack represents the observed count of the corresponding value. The variables with the highest proportions of patients answering with “I do not know” are Q01, Q04, Q09, Q17, Q25, Q29 and Q30 which related to costs.

On the average, the PTOPS-I was completed in 6 minutes, 49 seconds (SD = 2.43). Subjects experienced difficulty or needed assistance 168 times with a mean of 0.47 (SD = 1.09) clarifications for each questionnaire. In Figure 1 the distributions of the variables are displayed.

Following Roush and Sonstroem [21], the *Enhancers* and the *Location* domains have a positive connotation, whilst the *Detractor* and *Cost* domains have a negative one. Hence, high scores on the items in the first two domains, and low scores on the items in the last ones correspond to high levels of satisfaction. To make all the items coherent with their domain, the 5-points Likert-scale for items Q04, Q09, Q10, Q16, Q19, Q21, Q25, and Q28 was reversed (1 = 5, 2 = 4, 4 = 2, 5 = 1) so that the highest score (5) corresponds to the greatest satisfaction.

The items with the highest average rating were Q7, with an average of 4.57, Q2 with an average rating of 4.49, and Q23, with an average of 4.53 on the inverted scale. Items with the lowest average ratings were Q14, with a mean of 3.93, and Q29, with 3.51.

The variables with the highest proportions of patients answering with ‘I do not know’ are all related to costs.

For the study of the reliability and the validity, we performed two separate analyses:

– Analysis 1. To evaluate whether our results were consistent with the four domains identified by Roush and Sonstroem for the U.S. version of the questionnaire (“R-S- domains”).

– Analysis 2. To explore the possible existence of a different domain structure, specific to an Italian version of the PTOPS, and analyze the characteristics of these ‘Italian-based’ domains (“I-domains”).

Analysis 1. Analysis of the constructs identified by Roush and Sonstroem.

#### Reliability

**Internal consistency** The Cronbach’s α obtained for each of the four domains-totals of the PTOPS-I are shown in Table 2. This table also displays the values of α obtained after having deleted one item at a time from each domain. It clearly emerges from the data that some items are not particularly connected to the others: Q16 (‘I have to wait too long between appointments’) and Q29 (‘The facility appreciates my business’) in the R-S *Enhancers* domain; Q8 (‘It is difficult for me to get into the facility from the parking lot’) in the R-S *Detractors* domain; Q14 (‘The facility is in a desirable location’) in the R-S *Location* domain, and Q11 (‘I feel my therapist overcharges me’) and Q25 (‘My therapist does not expect me to pay significantly more than what my insurance covers’) in the R-S *Cost* domain. Moreover, the corrected items in total correlation show a general low-moderate α correlations. As shown by the figures in Table 2, the PTOPS-I exhibits a good overall reliability: the α values are acceptable for the *Enhancers* and *Cost* domains (α = 0.758 and 0.706 respectively), and are good for the *Detractors* and *Location* domains (α = 0.848 and 0.886 respectively).

**Table 2:** Reliability analysis with each question deleted (items arranged into the R-S-domains)

**Domain**	**Item**	**Cronbach's α if item deleted**	**Corrected item-total correlation**	**Overall Cronbach's α**
**Enhancers**	**Q02**	0.742	0.405	0.758
	**Q07**	0.733	0.481	
	**Q12**	0.725	0.510	
	***Q16*** *	**0.771**	**0.242**	
	**Q18**	0.725	0.510	
	**Q22**	0.725	0.517	
	**Q24**	0.740	0.410	
	**Q26**	0.731	0.477	
	**Q29**	**0.761**	**0.256**	
	**Q34**	0.726	0.505	
**Detractors**	**Q03**	0.831	0.576	0.848
	**Q06**	0.839	0.501	
	**Q08**	**0.863**	**0.300**	
	**Q13**	0.841	0.458	
	**Q15**	0.823	0.662	
	**Q20**	0.834	0.534	
	**Q23**	0.830	0.593	
	**Q27**	0.828	0.616	
	**Q31**	0.825	0.639	
	**Q33**	0.818	0.732	
**Location**	**Q05**	0.868	0.696	0.886
	***Q10*** *	0.876	0.650	
	**Q14**	**0.902**	**0.360**	
	***Q19*** *	0.856	0.777	
	***Q21*** *	0.857	0.773	
	***Q28*** *	0.863	0.723	
	**Q32**	0.856	0.778	
**Cost**	**Q01**	0.665	0.450	0.706
	**Q11**	**0.754**	**0.070**	
	***Q04*** *	0.653	0.496	
	***Q09*** *	0.615	0.635	
	**Q17**	0.621	0.589	
	**Q30**	0.629	0.575	
	***Q25*** *	**0.734**	**0.122**	

* To have consistency between the positive or negative connotation of its domain, the Likert-scale of the item has been reversed.

The most critical items within each domain are highlighted.

*Construct validity* was investigated by factor analysis of *each domain* separately. The proportion of total variance explained by the factor as well as its correlations (loadings) with the items are reported in Table 3. In particular the R-S *Locations* and *Detractors* domains are characterized by the strongest internal coherence (for these domains the first factor explains a substantial proportion of the total variance equal to 0.6 and 0.46 respectively, and medium to high correlations between the factor and the domains-items) whilst the factors extracted for the R-S *Enhancers* and *Costs* domains appear to be weaker (i.e., exhibited moderate proportions of explained total variance, equal to 0.34 and 0.4 respectively). By analyzing the factor loadings, and by increasing the number of extracted factors for each domain to explore whether more factors were necessary to satisfactorily describe the items assigned to each domain, we noted that the relatively poor performance of the first factors were substantially due to the presence of some variables that were weakly related to the others within the same domain. These variables were the same which impacted the analysis of Cronbach’s α’s. Hence, factor analysis substantially confirmed the results of the Cronbach’s α’s reported in Table 2, with respect both to the R-S- domains’ internal consistency and to the most critical variables within each R-S- domain.

**Table 3:** PTOPS items arranged into the R-S- domains

**Domain**	**Item**	**Label**	**Loading with Factor1**
**Enhancers**	**Q02**	I enjoy listening to my therapist	0.54
	**Q07**	I am given privacy when I need it	0.62
	**Q12**	The office staff is attentive to my needs	0.65
	***Q16*** *	I have to wait too long between appointments	0.35
	**Q18**	This facility is a nice place to get my therapy	0.65
	**Q22**	I can get around easily inside of the facility	0.66
	**Q24**	My therapist seems to have a genuine interest in me as a person	0.56
	**Q26**	I anticipate my questions will be answered clearly	0.63
	**Q29**	This facility appreciates my business	0.37
	**Q34**	I get along well with everyone in this PT facility	0.65
		**Variance explained**	**3.35**
		**Proportion of variance explained**	**0.34**
**Detractors**	**Q03**	I expect the facility to be quieter than it is	0.68
	**Q06**	I expect my therapist to spend more time with me than he/she does	0.61
	**Q08**	It is difficult for me to get into the facility from the parking lot	0.37
	**Q13**	My therapist acts like he/she is doing me a big favor by treating me	0.57
	**Q15**	My therapist could communicate with me more	0.76
	**Q20**	The facility is too crowded	0.64
	**Q23**	I don’t really enjoy talking with my therapist	0.71
	**Q27**	My therapist doesn’t give me a chance to say what is on my mind	0.73
	**Q31**	My therapist should be more thorough in my treatment	0.75
	**Q33**	My therapist should listen more carefully to what I tell him/her	0.83
		**Variance explained**	**4.57**
		**Proportion of variance explained**	**0.46**
**Location**	**Q05**	The distance required to get to the facility is acceptable to me	0.79
	***Q10*** *	This facility could be more conveniently located for me	0.74
	**Q14**	The facility is in a desirable location	0.47
	***Q19*** *	It is somewhat difficult to reach this PT facility	0.84
	***Q21*** *	I have to travel too far to receive my treatment	0.86
	***Q28*** *	I should not have to travel this far for therapy	0.82
	**Q32**	The physical therapy facility is conveniently located for me	0.85
		**Variance explained**	**4.22**
		**Proportion of variance explained**	**0.60**
**Cost**	**Q01**	The cost of treatment more than expected	0.65
	***Q04*** *	The facility is flexible about payment options	0.71
	***Q09*** *	I am charged a reasonable amount for my therapy	0.82
	**Q11**	I feel my therapist overcharges me	0.14
	**Q17**	The quality of the care I receive is not compatible with the cost	0.74
	***Q25*** *	My therapist does not expect me to pay significantly more than what my insurance covers	0.24
	**Q30**	It could be easier to make the arrangements to pay for my therapy	0.77
		**Variance explained**	**2.80**
		**Proportion of variance explained**	**0.40**

* To have consistency between the positive or negative connotation of its domain, the Likert-scale of the item has been reversed.

For each domain, factor analysis was performed and one factor extracted. The loading (correlation) of each item with its domain’s factor are reported.

#### Test-retest stability

To assess the repeatability of the PTOPS-I, the questionnaire was re-administered to a sample of 56 patients one week after the first filling. 53 out of the 56 subjects returned complete information. The relation between results gathered in the two administrations was investigated for each item and for each domain total using the ICC coefficients (see Table 4). Some coefficients appear not particularly high (around 0.5), even if they are all significantly higher than zero (as clearly indicated by the ICC 95% confidence intervals). Focusing on the domains sub-totals, the highest ICC was observed for the *Detractors* domain (with ICC = 0.891, and ICC 95% CI = 0.818-0.936), followed by the *Location* and *Cost* domains (both characterized by ICC = 0.860 and ICC 95% CI = 0.768-0.917) and by the *Enhancers* domain (with ICC = 0.766, and ICC 95% CI = 0.626-0.859).

**Table 4:** Intra-class Correlation Coefficients (ICC 3,1) for each item and for each R-S-domain (day 1 and day 7)

**Variable**	**N**	**ICC(3,1)**	**95% Confidence limits**	
**Q01**	53	0.785	0.653	0.870
**Q02**	53	0.583	0.371	0.737
**Q03**	53	0.648	0.457	0.781
**Q04**	53	0.936	0.891	0.963
**Q05**	53	0.638	0.444	0.775
**Q06**	53	0.514	0.283	0.688
**Q07**	53	0.754	0.607	0.851
**Q08**	53	0.799	0.675	0.880
**Q09**	53	0.862	0.772	0.918
**Q10**	53	0.558	0.338	0.719
**Q11**	53	0.546	0.324	0.712
**Q12**	53	0.449	0.203	0.641
**Q13**	53	0.926	0.875	0.957
**Q14**	53	0.651	0.463	0.784
**Q15**	53	0.872	0.788	0.925
**Q16**	53	0.466	0.225	0.654
**Q17**	53	0.828	0.718	0.897
**Q18**	53	0.324	0.059	0.546
**Q19**	53	0.645	0.454	0.779
**Q20**	53	0.547	0.325	0.712
**Q21**	53	0.921	0.867	0.954
**Q22**	53	0.567	0.350	0.726
**Q23**	53	0.522	0.293	0.694
**Q24**	53	0.660	0.475	0.790
**Q25**	53	0.816	0.699	0.890
**Q26**	53	0.630	0.434	0.770
**Q27**	53	0.641	0.448	0.777
**Q28**	53	0.760	0.616	0.855
**Q29**	53	0.576	0.362	0.732
**Q30**	53	0.726	0.567	0.833
**Q31**	53	0.833	0.726	0.900
**Q32**	53	0.736	0.581	0.839
**Q33**	53	0.841	0.739	0.906
**Q34**	53	0.899	0.830	0.940
**DOMAIN: ENHANCERS**	53	0.766	0.626	0.859
**DOMAIN: DETRACTORS**	53	0.891	0.818	0.936
**DOMAIN: LOCATION**	53	0.860	0.768	0.917
**DOMAIN: COST**	53	0.860	0.768	0.917

#### Validity

*Concurrent validity* was assessed by calculating the Pearson correlation coefficient between the PTOPS-I totals and the scores of the other administered questionnaires (GPE and VAS). As shown in Table 5, the GPE was significantly associated with the *Enhancers* (*r* = −0.429, *P* < 0.0001), *Detractors* (*r* = 0.281, *P* < 0.0001) and *Cost* (*r* = 0.328, *P* < 0.0001) totals, but was not related to the *Location* total. As for VAS, only the correlations with the *Enhancers* and the *Detractors* totals were statistically different from zero, but they demonstrated very low values.

**Table 5:** Concurrent validity analysis: Pearson Correlation Coefficients (p-value in parentheses)* between GPE and VAS and the R-S- domains total scores

	**Enhancers**	**Detractors**	**Location**	**Cost**
**GPE**	**−0.429** (<.0001)	**0.281** (<.0001)	−0.042 (0.429)	**0.382** (<.0001)
**VAS**	**−0.149** (0.005)	**0.164** (0.002)	−0.070 (0.194)	0.053 (0.328)

* Bolded coefficients are significantly different from zero.

Analysis 2. Determination of constructs possibly different from those identified by Roush and Sonstroem.

In the previous analysis, attention was focused on the domains determined by Roush and Sonstroem [21] on data collected on Italian patients. We then evaluated whether a different domain specification was more suited to our data. To accomplish the objectives of this aim, factor analysis was performed using the full set of items, using the principal components extraction method and the varimax rotation criterion.

We extracted 4 factors, explaining together 48.6% of the total variance. By analyzing the correlations (loadings) between the items and the four factors (see Table 6), and by assigning each item to the factor to which it was most correlated, we observed a distribution of items across four factors that were similar to Roush and Sonstroem with some interesting differences. More precisely, item Q16 (‘I have to wait too long between appointments’) which was placed into the R-S *Enhancers* domain and item Q11 (‘I feel my therapist overcharges me’) assigned to the R-S *Cost* domain were instead included in our first I-domain (mostly overlapping with the R-S *Detractors* domain); item Q14 (‘The facility is in a desirable location’) assigned to the *Location* R-S domain was instead associated with the third I-domain (mostly overlapping with the R-S *Enhancers* domain).

**Table 6:** PTOPS items arranged into the Italian-based domains and results of the factor analysis (principal components extraction method, and varimax rotation criterion)

**Item**		Factor loadings*			
		**1**	**2**	**3**	**4**
**Q33**	My therapist should listen more carefully to what I tell him/her	0.79	0.11	−0.12	.
**Q15**	My therapist could communicate with me more	0.76	.	−0.16	.
**Q27**	My therapist doesn’t give me a chance to say what is on my mind	0.72	.	−0.19	.
**Q11**	I feel my therapist overcharges me	0.69	.	−0.11	.
**Q23**	I don’t really enjoy talking with my therapist	0.67	.	.	.
**Q31**	My therapist should be more thorough in my treatment	0.66	.	−0.11	0.11
**Q03**	I expect the facility to be quieter than it is	0.62	.	−0.19	.
**Q20**	The facility is too crowded	0.60	0.14	−0.15	.
**Q16**	I have to wait too long between appointments	0.59	.	.	0.17
**Q13**	My therapist acts like he/she is doing me a big favor by treating me	0.58	0.13	−0.13	−0.25
**Q06**	I expect my therapist to spend more time with me than he/she does	0.53	.	−0.17	.
**Q19**	It is somewhat difficult to reach this PT facility	0.19	0.83	−0.11	.
**Q21**	I have to travel too far to receive my treatment	0.24	0.82	.	.
**Q28**	I should not have to travel this far for therapy	0.31	0.78	.	.
**Q10**	This facility could be more conveniently located for me	.	0.75	.	.
**Q05**	The distance required to get to the facility is acceptable to me	.	−0.79	0.16	.
**Q32**	The physical therapy facility is conveniently located for me	.	−0.84	0.23	.
**Q18**	This facility is a nice place to get my therapy	.	−0.17	0.69	−0.15
**Q34**	I get along well with everyone in this PT facility	.	−0.11	0.64	.
**Q22**	I can get around easily inside of the facility	−0.19	−0.14	0.63	.
**Q26**	I anticipate my questions will be answered clearly	−0.18	.	0.60	0.13
**Q12**	The office staff is attentive to my needs	−0.27	.	0.57	−0.11
**Q24**	My therapist seems to have a genuine interest in me as a person	−0.24	0.16	0.54	.
**Q14**	The facility is in a desirable location	.	−0.38	0.52	.
**Q07**	I am given privacy when I need it	−0.31	.	0.52	−0.15
**Q02**	I enjoy listening to my therapist	−0.21	.	0.46	−0.12
**Q29**	This facility appreciates my business	0.13	.	0.39	−0.37
**Q30**	It could be easier to make the arrangements to pay for my therapy	0.17	.	.	0.75
**Q17**	The quality of the care I receive is not compatible with the cost	0.33	.	.	0.68
**Q01**	The cost of treatment more than expected	0.14	.	.	0.60
**Q08**	It is difficult for me to get into the facility from the parking lot	0.32	0.21	.	0.42
**Q25**	My therapist does not expect me to pay significantly more than what my insurance covers	.	0.20	.	−0.21
**Q04**	The facility is flexible about payment options	0.13	.	.	−0.71
**Q09**	I am charged a reasonable amount for my therapy	.	.	0.12	−0.80
	**Variance explained by the factor**	**5.633**	**4.269**	**3.492**	**3.138**
	**Proportion of explained variance**	**0.166**	**0.126**	**0.103**	**0.092**

*Loadings lower than 0.1 (in absolute value) are not printed.

Also, our results suggested that item Q08 (‘It is difficult for me to get into the facility from the parking lot’) had to be assigned to the fourth I-domain, since surprisingly it showed the highest correlation with the fourth factor (summarizing the *Cost*-items), even if the loading was not particularly high (0.42). Nonetheless, a weaker relation (*r* = 0.32) was also observed with the first factor. From the results reported in Table 3 one can note that Q08 is not particularly related to the other items placed in the R-S *Detractors* domain. Also, the correlation (loading) with the unique factor extracted to evaluate the construct validity of the R-S *Detractors’* domain was rather low (0.37) and aligned with the correlation observed with the first I-factor (0.32). Based on these considerations, for the sake of interpretation, we assigned the item to the first I-domain. Therefore, we determined that the R-S factor names were not completely relevant to an Italian context and chose *‘Depersonalization’, ‘Inaccessibility’, ‘Ambience’*, and *‘Cost’* to name the domains corresponding to the four I-factors.

The internal consistency of the I-domains is higher compared to the R-S domains. The overall Cronbach’s alpha reaches 0.784 for the *Ambience* I-domain, 0.87 for the *Depersonalization* I-domain, and 0.902 and 0.754 for the *Inaccessibility* and the *Cost* I-domains respectively.

Construct validity was again analyzed by applying four factor analyses, one for each I-block. The obtained results (displayed in Table 7) are very similar to those observed for the R-S- domains, and also in this case are consistent with the indications provided by the Cronbach’s α’s.

**Table 7:** PTOPS items arranged into the I-domains

**Depersonalization**		**Factor1**
**Q33**	My therapist should listen more carefully to what I tell him/her	0.813
**Q15**	My therapist could communicate with me more	0.774
**Q27**	My therapist doesn’t give me a chance to say what is on my mind	0.733
**Q11**	I feel my therapist overcharges me	0.689
**Q23**	I don’t really enjoy talking with my therapist	0.683
**Q31**	My therapist should be more thorough in my treatment	0.696
**Q03**	I expect the facility to be quieter than it is	0.657
**Q20**	The facility is too crowded	0.650
**Q16**	I have to wait too long between appointments	0.617
**Q13**	My therapist acts like he/she is doing me a big favor by treating me	0.571
**Q06**	I expect my therapist to spend more time with me than he/she does	0.577
**Q08**	It is difficult for me to get into the facility from the parking lot	0.377
	**Variance explained**	**5.257**
	**Proportion of variance explained**	**0.438**
**Inaccessibility**		**Factor1**
Q05*	The distance required to get to the facility is acceptable to me	0.785
**Q10**	This facility could be more conveniently located for me	0.759
**Q19**	It is somewhat difficult to reach this PT facility	0.863
**Q21**	I have to travel too far to receive my treatment	0.859
**Q28**	I should not have to travel this far for therapy	0.824
Q32*	The physical therapy facility is conveniently located for me	0.848
	**Variance explained**	**4.072**
	**Proportion of variance explained**	**0.679**
**Ambience**		**Factor1**
**Q02**	I enjoy listening to my therapist	0.510
**Q07**	I am given privacy when I need it	0.602
**Q12**	The office staff is attentive to my needs	0.643
**Q18**	This facility is a nice place to get my therapy	0.707
**Q22**	I can get around easily inside of the facility	0.659
**Q24**	My therapist seems to have a genuine interest in me as a person	0.532
**Q26**	I anticipate my questions will be answered clearly	0.615
**Q29**	This facility appreciates my business	0.379
**Q34**	I get along well with everyone in this PT facility	0.641
**Q14**	The facility is in a desirable location	0.554
	**Variance explained**	**3.494**
	**Proportion of variance explained**	**0.349**
**Cost**		**Factor1**
**Q01**	The cost of treatment more than expected	0.641
Q04*	The facility is flexible about payment options	0.716
Q09*	I am charged a reasonable amount for my therapy	0.821
**Q17**	The quality of the care I receive is not compatible with the cost	0.731
Q25*	My therapist does not expect me to pay significantly more than what my insurance covers	0.258
**Q30**	It could be easier to make the arrangements to pay for my therapy	0.764
	**Variance explained**	**2.781**
	**Proportion of variance explained**	**0.463**

For each domain, factor analysis was applied and one factor extracted. For each item, its loading (correlation) with its domain’s factor are reported.

#### Test-retest stability

Results are similar to those obtained for the Roush and Sonstroem blocks: as for the total scores (obtained by summing up the scores of all the items in each block) the ICC’s turned out to be 0.745 (95%C.I.: 0.596-0.846) for *Ambience*, 0.903 (95%C.I.: 0.835-0.942) for *Depersonalization*, 0.875 (95%C.I.: 0.789-0.925) for *Location*, and 0.855 (95%C.I.: 0.758-0.913) for *Cost*.

#### Concurrent validity

As shown in Table 8, GPE is significantly associated only to the totals based on *Depersonalization* (*r* = 0.269, *P* < 0.001), *Ambience* (*r* = −0.378, *P* < 0.001)*,* and *Cost*, (*r* = 0.356, *P* < 0.001) but not related to the *Inaccessibility* total. VAS is not significantly related to the considered totals. Even though some correlations have relatively low p-values, very low values of the coefficients were also observed.

**Table 8:** Concurrent validity analysis: Pearson Correlation Coefficients (p-value in parentheses)* between GPE and VAS and the I-domains total scores

	**Ambience**	**Depersonalization**	**Inaccessibility**	**Cost**
**GPE**	**−0.378** (<.0001)	**0.269** (<.0001)	−0.015 (0.779)	**0.356** (<.0001)
**VAS**	−0.090 (0.099)	*0.131* (0.016)	0.061 (0.264)	0.030 (0. 587)

* Bolded coefficients are significantly different from zero; figures in italics are significant at the 2.5% level.

To evaluate the relative importance of the subtotals on the global level of satisfaction, a total score was obtained by summing up the scores on all the items, recoding some variables for analytic purposes to ensure that all the items had the same positive direction. The 4 totals (*Depersonalization*, *Inaccessibility*, *Ambience*, *Cost*) were also re-built using the modified item blocks. The correlations between the total score and the four sub-totals showed that the *Depersonalization* total is the one most correlated to the global one (*r* = 0.828, *P* < 0.0001), followed by *Ambience* (*r* = 0.707, *P* < 0.0001), *Inaccessibility* (*r* = 0.619, *P* < 0.0001), and *Cost* (*r* = 0.361, *P* < 0.0001). Note that the low impact of the last domain on the grand total is expected since the *Cost*-items are those characterized by the highest proportion of ‘I do not know’ responses. To evaluate the impact of each sub-total on the grand total accounting for the other sub-totals, the grand-total was regressed on the three most relevant sub-totals using a linear model. Our results confirmed that the *Depersonalization* total (and hence the items it comprises) most influences global level of satisfaction.

#### Analysis of the dependency of the indicators of satisfaction

Finally, we analyzed the dependency of satisfaction on the background variables, i.e. the variables related to the characteristics of the facility and/or of the therapist and of the patients. Because these variables are categorical, we used an ANOVA procedure, testing the null hypothesis that the means do not vary according to the levels of each explanatory variable for each sub-total. Since the distribution of the sub-totals was found not to be normal, a non-parametric approach was used, based on the Kruskal-Wallis or on the Wilcoxon test for explanatory variables having only two levels. For the sake of a compact synthesis of results, only the most relevant results are reported here.

These analyses determined differences in *Ambience* and *Cost* sub-totals across the three facilities (*P* = 0.0015 for *Ambience*; *P* < 0.0001 for *Cost*). Furthermore, we observed that for all the sub-totals but *Location,* the most satisfied patients are those having a female therapist (*P* = 0.015 for *Depersonalisation*, *P* < 0.0001 for *Ambience*, *P* = 0.0021 for *Cost*) and those who regularly attend the facility (*P* = 0.007 for *Depersonalisation*, *P* =0.004 for *Ambience*, *P* = 0.0005 for *Cost)*. The interaction between the patient’s and the therapist’s gender was not significant. Moreover, all the sub-totals but not *Depersonalisation* differ in mean across the age strata and education level (*P* = <.0001 and *P* = 0.0036 for *Ambience*, *P* = 0.0055 and *P* = 0.0313 for *Location*, *P* = 0.0087 and *P* = 0.0086 for *Cost*). The most satisfied patients were the oldest ones and those with the lowest level of education ones. These findings are related as the oldest persons in our sample were those with the lowest levels of education. Finally, only for the *Ambience* sub-total, a significant difference in means was observed across the working status (*P* < .0001), the retired and the unemployed patients being the most satisfied ones.

## Discussion

The Italian version of the Physical Therapy Outpatient Satisfaction Survey (PTOPS-I) showed a good level of patient acceptability and required only few minutes to complete. A high number of responses ‘I do not know’ were noted for items related to cost. A possible explanation for this result can be found in the different social and economic organizations of the U.S. and Italy. In Italy, patients can have access to completely free medical care depending on their clinical or social situation. In other cases, physical therapy treatments are reimbursed by private insurance companies, and yet in still other cases, patients pay themselves for the treatment they receive.

Patients who have access to a public service and do not pay for it may have very different expectations compared with private patients who pay for the treatments themselves, and with patients who get treatments reimbursed by their own private medical insurance.

Furthermore, in Italy public facilities have long waiting lists of patients, so there may be fewer incentive to address patient satisfaction as reward systems are usually set by ‘political’ or administrative decisions. In contrast, satisfaction with the outcome of care for each single patient can function as a strong economic incentive for an independent private physical therapist.

Despite the high proportion of ‘I do not know’ responses on *Cost* variables, we analyzed the PTOPS-I questionnaire without any type of modification on these items to preserve our intent to conduct a comparison with the original instrument and its factor structure which included R-S *Cost*. Based on the data supporting the R-S- domains, there was substantial indication that the inclusion of these items in the analysis would not influence examination of the other factors. In fact, when factor analysis was applied to the Italian data, the *Cost-*items again appeared to be related only to each other and showed a low level of correlation with the other items. Thus, including these items does not change our results.

Our first analysis, applying the same constructs identified by Roush and Sonstroem, showed a strongest internal coherence of the R-S *Locations* and *Detractors* domains and a weaker coherence of *Enhancers* and *Costs* domains. The performances of the factors were substantially due to the presence of some variables weakly related to the others within the same domain. These variables were the same which impacted the analysis of Cronbach’s α’s. Hence, factor analysis substantially confirmed the results of the Cronbach’s α’s, with respect both to the R-S- domains’ internal consistency and to the most critical variables within each R-S- domain.

The results of our concurrent validity analysis indicated that the items in the R-S *Location* domains are important but not particularly related to level of satisfaction in our sample.

A specific factor analysis applied to the Italian PTOPS data provided a clear structure of the factor loading matrix with only a few exceptions. For this reason, we decided to maintain a 4 factors structure in the Italian version. However, the I-domains are slightly different from the R-S ones, and therefore were given new factor names to capture the construct underlying the factor more aptly.

In Analysis 2 we observed that the items that changed domains on data collected from Italian patients turned out to be among the most critical ones in terms of their degree of association with the R-S- domains. These variables are more connected with the I-domains to which they are assigned, supporting the conclusion that the reassignment of the items into the I-domains more aptly captured the Italian data.

Internal consistency of the I-domains is higher compared to the R-S- domains: this is reasonable and largely expected, since the I- blocks were derived based on Italian data and not by extending the structure derived from the U.S. patients to an Italian population.

In our sample patient satisfaction was mostly related to the elements of *Depersonalization* (e.g., confidence, dialogue, respect for privacy), consistent with the findings of Beattie and colleagues [11], Hills and Kitchen [22] and Hush [23]. The lowest level of satisfaction was found on the *Cost* I-domain, replicating findings from the American study. From the analysis of the correlations between the total score and the four sub-totals, the *Depersonalization* domain appeared the most correlated to the global score. However, it is important to emphasize that patients tended to assign the highest (or the lowest) scores when responding to questionnaire items. As a consequence, small variations in the opinions had a very large effect on the Pearson correlation coefficient.

Based on both analysis, we found moderate correlations between the PTOPS-I totals and the GPE, yet correlations with the VAS score turned out to be very low or not significant. These results are consistent with the results of Kelly [24], Skolasky et al. [25] and with the systematic review of Hush and colleagues [23], which concluded that the relationship between satisfaction and clinical outcome is weak. George and Hirsh [26] showed that patients’ satisfaction with their treatment is independent of satisfaction with the outcome of the physical therapy.

The overall magnitude and direction of associations and factor loading were similar between our Italian version of the PTOPS and the American- and the European-English versions. Casserley-Feeney calculated the correlations between the four R-S- domains and 4 overall satisfaction indicators. One of these, defined as the ‘Overall improvement due to physiotherapy’, is comparable to the Global Perceived Effect used in our study. Thus, broad comparisons of patient satisfaction and overall improvement across cultures using data collected on translated instruments may be possible. The most substantial differences were observed for the I-*Cost* domain, which is the one with the highest percentage of ‘I don’t know’ responses. Thus, the contribution of ‘cost’ to patient satisfaction may always need to be interpreted with respect to the particular socioeconomic and cultural context in which the study was conducted.

### Limits

Some analyses were limited by the high number of ‘I don’t know’ responses (19 on items in two factors), largely due to differences in payment for health care between the United States and Italy. The analysis of the concurrent validity was made only with GPE and VAS as other relevant measures were not available in Italian. It was not possible to conduct additional analyses of construct validity as no other Italian-language questionnaire with the same constructs was available.

Test-retest values were lower than expected. This could depend on the timing of the evaluation, and therefore reflect a real change in opinion rather than an unsatisfying reliability of the instrument.

Questions related to the costs are pertinent in Italy only to patients who pay for treatment themselves. In the Italian version, *Cost* variables were not related to satisfaction, so these items might be deleted. However, it would be interesting to analyze whether patients have a different perception of the quality of treatment they received, depending on their expectations and on the method of payment. Thus, wording of specific cost-related items should be considered in future studies. A preference-based instrument could be more appropriate to measure satisfaction across different settings in a mixed system such as the Italian one.

Further studies using PTOPS-I are suggested to further investigate construct validity. Satisfaction could be investigated in subjects stratified by pathological conditions (e.g., neurologic, musculoskeletal, etc.) or degree of disability, so that the relationship among these variables and satisfaction could be deepened.

## Conclusions

This study describes the development of a translation, cultural adaptation, reliability and validity study of the Italian version of PTOPS. This study shows that the PTOPS, translated into Italian in its full version, is easy to understand and be self-administered, requiring only few minutes to be completed. It has good psychometric properties (internal consistency and test-retest stability) and its use can be recommended to evaluate patient satisfaction with outpatient physical therapy in different Italian health care facilities.

## Institutional Review Board approval of the study protocol

Ethics Committee of the University Hospital S. Orsola-Malpighi of Bologna (Italy) - code 32/2011/U/OssN.

## Competing interests

The authors declare that they have no competing interests.

## Authors’ contributions

CV and PP designed the study. FB and DC were responsible for data collection. RP was responsible for data analysis, together with AG, CV, FSV and PP contributed to interpretation of data, together with CV and FSV. CV, PP, RP and AG drafted the manuscript, together with FB and DC. All authors critically revised the manuscript. All authors read and approved the final manuscript.

## Pre-publication history

The pre-publication history for this paper can be accessed here:

http://www.biomedcentral.com/1471-2474/14/125/prepub

## Supplementary Material

Additional file 1 PTOPS–I Physical Therapy Outpatient Satisfaction Survey - Italian Version. Click here for file
