# Supplementary material for: Cross-cultural adaptation and validation of the Physical Therapy Outpatient Satisfaction Survey in an Italian musculoskeletal population
Source: BMC Musculoskelet Disord. 2013 Apr 5;14:125. doi: 10.1186/1471-2474-14-125 (PMC3623747; doi:10.1186/1471-2474-14-125)
Supplement: Additional file 1 — PTOPS–I Physical Therapy Outpatient Satisfaction Survey - Italian Version. [file 1471-2474-14-125-S1.pdf]

PTOPS-I (Physical Therapy Outpatient Satisfaction Survey - Versione Italiana)

Gentile paziente,
con il questionario che segue la invitiamo ad esprimere il suo parere rispetto al servizio di fisioterapia da noi offerto.
Le chiediamo quindi di dedicare alcuni minuti a compilare e restituire il questionario.
Inserisca una X nell'apposita casella, per indicare il suo giudizio. Le diamo la garanzia che ogni sua valutazione resterà anonima, non influenzerà in alcun modo il trattamento a lei erogato e che il suo fisioterapista non sarà informato su come lei avrà risposto.
Le saremo molto grati se ci restituirà il questionario il prima possibile.

	Molto in disaccordo	In disaccordo	Non so	D'accordo	Molto d'accordo
1. Il costo del trattamento è maggiore di quello che mi aspettavo.	1	2	3	4	5
2. Mi fa piacere seguire i consigli del mio fisioterapista.	1	2	3	4	5
3. Mi aspettavo che la struttura fosse più tranquilla.	1	2	3	4	5
4. La struttura è flessibile rispetto alle modalità di pagamento.	1	2	3	4	5
5. La distanza per raggiungere la struttura è accettabile per me.	1	2	3	4	5
6. Mi aspetto che il mio fisioterapista passi più tempo con me rispetto a quanto in realtà faccia.	1	2	3	4	5
7. La mia riservatezza viene rispettata.	1	2	3	4	5
8. È difficile raggiungere la struttura dal parcheggio.	1	2	3	4	5
9. Il costo del mio trattamento è ragionevole.	1	2	3	4	5
10. La struttura potrebbe essere in una posizione più comoda per me.	1	2	3	4	5
11. Penso che il mio fisioterapista mi sovraccarichi di lavoro.	1	2	3	4	5
12. Il personale della struttura è attento alle mie esigenze.	1	2	3	4	5
13. Il mio fisioterapista agisce come se stesse facendo un grosso favore nel trattarmi.	1	2	3	4	5
14. La struttura è in una posizione gradevole.	1	2	3	4	5
15. Il mio fisioterapista potrebbe comunicare meglio con me.	1	2	3	4	5
16. Devo aspettare troppo tempo tra un appuntamento e l'altro.	1	2	3	4	5
17. La qualità delle cure che ricevo non è adeguata ai costi.	1	2	3	4	5
18. La struttura è un posto gradevole dove fare la terapia.	1	2	3	4	5
19. E' un po' difficile per me raggiungere questa struttura di fisioterapia.	1	2	3	4	5
20. La struttura è troppo affollata.	1	2	3	4	5
21. Devo affrontare un viaggio troppo lungo per ricevere il mio trattamento.	1	2	3	4	5
22. Posso muovermi agevolmente all'interno della struttura.	1	2	3	4	5
23. Non mi piace parlare con il mio fisioterapista.	1	2	3	4	5
24. Il mio fisioterapista sembra interessarsi realmente a me come persona.	1	2	3	4	5
25. Il mio fisioterapista non richiede un pagamento superiore alla copertura assicurativa.	1	2	3	4	5
26. Mi aspetto che le mie domande abbiano una risposta chiara.	1	2	3	4	5
27. Il mio fisioterapista non mi dà la possibilità di dire ciò che ho in mente.	1	2	3	4	5
28. Non dovrei andare così lontano per il mio trattamento.	1	2	3	4	5
29. La struttura apprezza che io l'abbia scelta per il mio trattamento.	1	2	3	4	5
30. Dovrebbe essere più semplice mettersi d'accordo sulle modalità di pagamento del trattamento.	1	2	3	4	5
31. Il mio fisioterapista dovrebbe essere più accurato nel mio trattamento.	1	2	3	4	5
32. La struttura di fisioterapia è in una posizione comoda per me.	1	2	3	4	5
33. Il mio fisioterapista dovrebbe ascoltare più attentamente quello che gli dico.	1	2	3	4	5
34. Ho un buon rapporto con tutti in questa struttura di fisioterapia.	1	2	3	4	5
